# Optimization of the second internal transcribed spacer (*ITS2*) for characterizing land plants from soil

**DOI:** 10.1371/journal.pone.0231436

**Published:** 2020-04-16

**Authors:** Emma K. Timpano, Melissa K. R. Scheible, Kelly A. Meiklejohn

**Affiliations:** Department of Population Health and Pathobiology, North Carolina State University, College of Veterinary Medicine, Raleigh, North Carolina, United States of America; University of Helsinki, FINLAND

## Abstract

Molecular-based taxonomy, specifically DNA barcoding, has streamlined organism identification. For land plants, the recommended 2-locus barcode of *rbcL* and *matK* is not suitable for all groups, thus the second subunit of the nuclear internal transcribed spacer (*ITS2*) has received attention as a possible alternative. To date, evaluations of *ITS2* have mostly been limited in scope to specific plant orders/families and single source material. Prior to using *ITS2* to routinely characterize land plants present in environmental samples (*i*.*e*., DNA metabarcoding), a wet lab protocol optimized for bulk sample types is needed. To address this gap, in this study we determined the broad recoverability across land plants when using published *ITS2* primer pairs, and subsequently optimized the PCR reaction constituents and cycling conditions for the best two performing primer pairs (ITS2F/ITSp4 and ITSp3/ITSu4). Using these conditions, both primer pairs were used to characterize land plants present in 17 diverse soils collected from across the US. The resulting PCR amplicons were prepared into libraries and pooled for sequencing on an Illumina^®^ MiniSeq. Our existing bioinformatics workflow was used to process raw sequencing data and taxonomically assign unique *ITS2* plant sequences by comparison to GenBank. Given strict quality criteria were imposed on sequences for inclusion in data analysis, only 43.6% and 7.5% of sequences from ITS2F/ITSp4 and ITSp3/ITSu4 respectively remained for taxonomic comparisons; ~7–11% of sequences originated from fungal co-amplification. The number of orders and families recovered did differ between primer pairs, with ITS2F/ITSp4 consistently outperforming ITSp3/ITSu4 by >15%. Primer pair bias was observed in the recovery of certain taxonomic groups; ITS2F/ITSp4 preferentially recovered flowering plants and grasses, whereas ITSp3/ITSu4 recovered more moss taxa. To maximize data recovery and reduce potential bias, we advocate that studies using *ITS2* to characterize land plants from environmental samples such as soil use a multiple primer pair approach.

## Introduction

It is common practice in the scientific community to harness the information contained within short, yet informative, regions of the genome to molecularly discriminate between species. This approach, commonly known as DNA barcoding, has been applied globally to organisms across the tree of life, and has reduced the burden on taxonomic experts for species identifications. In traditional DNA barcoding, the appropriate barcode region is amplified from a single individual and the resulting sequence is identified by comparing it to a reference library of barcode sequences. With advances in sequencing technologies (*i*.*e*., next generation sequencing [NGS]), characterizing the entire biological community present in bulk environmental samples such as soil, water, air, dust and feces (known as DNA metabarcoding) has gained momentum.

Over a decade ago, the Consortium for the Barcode of Life (CBOL) Plant Working Group broadly assessed the utility of seven plastid DNA regions for discriminating amongst land plants [1]. Their recommendation was a 2-locus combination, *rbcL* and *matK*, which permitted identification to the species level in 72% of cases [1]. They did however acknowledge that alternate loci might be needed to augment identification in some groups, especially as routine amplification of *matK* can be difficult and *rbcL* can often only permit discrimination to the genus level or higher [1].

Two plastid regions, the intergenic spacer *trnH-psbA* and the P6 loop of the *trn*L (UAA) intron, are often employed as supplemental barcoding loci as they can be routinely amplified across land plants, permit species-level resolution, and recovery is possible even from highly degraded samples (*e*.*g*., [1–6]). However, the most commonly sequenced supplementary locus is the internal transcribed spacer (*ITS*) of the nuclear ribosomal cistron (18S-5.8S-26S) (*e*.*g*., [4,7–9]). Primers complementary to the flanking rRNA genes facilitate straight-forward amplification of the two highly variable subunits (*ITS1* and *ITS2*), and large public databases of *ITS* sequences already exist to facilitate the identification of unknowns (*e*.*g*., GenBank, BOLD). Use of *ITS* for species-level identification is especially advantageous in taxa which have lost their plastid genomes (*e*.*g*., holoparasitic plants, green algae; [10,11]). Despite its utility, three main disadvantages have hindered the broad adoption of *ITS* as the primary supplemental barcoding locus for discrimination amongst land plants: 1) the most commonly used primer pair [12] permits co-amplification of fungal DNA if present, leading to misidentifications, 2) multiple and possibly divergent copies of *ITS* can exist in a single individual, which impedes the recovery of clean sequence data using Sanger technology, and 3) for highly degraded samples, recovery of the complete *ITS* region (up to 3,000 bp in some *Pinus* species; [13]) is challenging [3,14].

To address these perceived shortcomings, recent studies have focused on the utility of only *ITS2* as both a supplemental and stand-alone barcode locus for land plants. In spite of its short length (160–390 bp), *ITS2* possesses high interspecific divergence allowing at least genus-level but in most cases also species-level identification across all land plants (*e*.*g*., [4,7,15–17]). To alleviate concerns with co-amplification of fungal DNA, several studies have designed, tested and published plant specific *ITS2* primers [7,16,17]. While high specificity for these *ITS2* primers have been reported, the majority of previous studies completed evaluations in specific taxonomic groups with single source material. Given the growing interest in using DNA metabarcoding to conduct surveys of biological communities, there is a need to assess the utility and applicability of *ITS2* for characterizing plants in bulk sample types.

The aims of this study were to: 1) identify plant specific *ITS2* primer pairs from the scientific literature, and determine their broad utility across diverse land plants, 2) optimize the PCR reaction components and cycling conditions for the best performing *ITS2* primer pairs, 3) characterize land plants present in diverse soils using the optimized *ITS2* conditions and NGS, and 4) establish the specificity, recoverability and any bias in the plants recovered from soils using *ITS2*.

## Materials and methods

### Samples

To ensure optimized conditions worked broadly across land plants, a single representative from the four predominant plant groups was used: angiosperm (*Gerbera* sp.), fern (*Nephrolepis* sp.), gymnosperm (*Pinus* sp.) and moss (*Entodon* sp.). Fresh material from these four species was either sourced from Raleigh, NC (moss, pine, fern) or purchased from a florist (angiosperm). Given the enormous diversity of angiosperms (>350,000 species; [3]), dried herbarium material was sourced from the National Museum of Natural History (USNM; Washington, DC) for an additional seven taxa spanning six orders: *Erythropleum africanum* (Fabales: Fabaceae), *Erythrina crista-galli* (Fabales: Fabaceae), *Acinitum napellus* (Ranunculales: Ranunculaceae), *Croton tiglium* (Malpighiales: Euphorbiaceae), *Hyoscyamus niger* (Solanales: Solanaceae), *Conium maculatum* (Apiales: Apiaceae). The optimized protocol was tested using 17 diverse soils, which were collected between 2007–2010 as part of the U.S. Geological Survey (USGS) geochemical and mineralogical survey of soils from the conterminous US. [18]. Briefly, collected soil from each study site was allowed to air-dry at ambient temperature, disaggregated and sieved (<2 mm), and the remaining material stored in a low temperature and humidity environment in the USGS archive (Denver, CO). In this study, a subsample of the A horizon (the uppermost mineral soil) was obtained for downstream *ITS2* DNA metabarcoding. Information on the specific collection location for these soils along with attributes are given in S1 Table.

### DNA isolation and quantification

The DNeasy Plant Mini Kit (Qiagen, Hilden, Germany) was used to isolate the total genomic DNA from plant samples. To ensure cell lysis, plant material was initially ground using Kimble Tissue Homogenizers (Kimble Chase, Vineland, NJ). A total of 100 mg of surface soil was used as input for DNA extractions using the PowerSoil Kit (Qiagen). The manufacturers protocols were followed for both kits with only a single deviation; DNA was eluted using either 50 μL (moss, angiosperm, fern and soil samples) or 25 μL (gymnosperm) of buffer to increase the final concentration. DNA was kept in LoBind tubes (Eppendorf, Hamburg, Germany) at 4°C while in use and stored at -20°C. The total genomic DNA yielded from both plant and soil extractions was quantified using the Qubit^™^ 3 Fluorometer (Invitrogen, Carlsbad, CA) and the Qubit^™^ HS DNA Assay Kit (Invitrogen). Reagent blanks were carried through the extraction process and as they were free of DNA (as determined using the Qubit), they were not carried through the remainder of the workflow.

### Primer pair screening

From the published literature, four primer pairs for the second internal transcribed spacer of ribosomal DNA (*ITS2*) were selected for screening as they 1) amplify a region <500 bp, making them amenable for sequencing on most NGS platforms, and 2) had been reported to amplify robustly across the four predominant land plant groups. All amplifications were performed on a Veriti^™^ 96-well Thermal Cycler (Applied Biosystems^™^, Foster City, CA) using the KAPA3G Plant DNA polymerase (KAPA Biosystems, Wilmington, MA). The KAPA Plant DNA polymerase was chosen as it is reported by the manufacturer to have a tolerance to PCR-inhibitors such as polyphenols, which are common in soil. Each 25 μL reaction mix consisted of 12.5 μL of 2X KAPA Plant PCR buffer (final concentration 1X), 0.5 U of KAPA3G Plant DNA polymerase, 0.3 uM of each primer and 2 μL of either DNA (angiosperm, fern, gymnosperm, moss or soil), or nuclease free water (negative control). The published cycling conditions for each primer pair were implemented for screening (S2 Table). To confirm whether each primer pair was cleanly amplifying *ITS2*, amplicons were visualized via gel electrophoresis using the Lonza FlashGel^®^ System (Lonza, Rockland, ME) and 2.2% agarose FlashGel^®^ DNA cassettes (Lonza).

### Primer optimization

For the two best performing primer pairs, two approaches were taken to reduce the amplification of secondary products. Firstly, the impact of shortening the extension time to 5, 10, or 20 seconds was assessed. Secondly, the effectiveness of adding dimethyl sulfoxide (DMSO) to the reaction mix at final concentrations previously reported as useful (*i*.*e*., 1%, 3%, 3.5%, 4% and 5% [19]) was examined. In these optimization experiments, the five positive control DNA samples (angiosperm, fern, gymnosperm, moss and soil) were included in each PCR. After identifying the optimal PCR constituents and cycling conditions for each primer pair, the optimized conditions were more broadly tested on the additional seven plant taxa sourced from the USNM. All generated amplicons were visualized via gel electrophoresis as described above.

### Testing and sequencing with bulk surface soil samples

Using the optimized PCR constituents and cycling conditions for each primer pair, *ITS2* was amplified in duplicate from the 17 soils sourced from the USGS archive. A pooled positive, which consisted of equal volumes of ~0.5 ng/μL dilutions of the individual positive control plant DNAs (*i*.*e*., angiosperm, fern, gymnosperm, moss) was used, and the negative control consisted of nuclease-free water. Generated amplicons were purified with 1.8 volumes of Agencourt AMPure XP Reagent (Beckman Coulter, Brea, CA), and subsequently eluted in 60 μL of Buffer EB (Qiagen). For quality control, 2 μL was used to measure DNA quantity with the Qubit dsDNA HS Assay (Thermo Fisher Scientific, Waltham, MA), and 5 μL was used to verify product on a FlashGel DNA cassette (Lonza). A total of 50 μL of each purified amplicon was used as input for library preparation using the KAPA Hyper Prep Kit (KAPA Biosystems). Following the manufacturer’s protocol, libraries were prepared with compatible Illumina^®^ indices/adapters and eluted in 25 μL of Buffer EB (Qiagen) following purification. Each library was individually quantified using the KAPA Library Quantification kit (KAPA Biosystems), and an appropriate volume of each library (n, 38) was combined in a single LoBind tube (Eppendorf) to create an approximately equi-molar library pool. The concentration of the final library pool was verified using the KAPA Library Quantification kit (KAPA Biosystems). The pooled library was prepared as described in the Illumina^®^ Denature and Dilute Libraries Guide (Document # 15039740 v10) with a 50% PhiX spike and final loading concentration of 1.4 pM. This pool was subsequently sequenced on a single run of the Illumina^®^ MiniSeq using the MiniSeq System Mid-Output Kit (Illumina^®^, San Diego, CA; 1 X 300 bp).

### Sequence data analysis

Raw sequence data was processed and analyzed following the pipeline described in [20]. Briefly, raw reads were filtered and trimmed using the default parameters of the DADA2 pipeline [21]. Primer sequences were trimmed from the unique sequences identified using DADA2, and duplicate sequences identified and excluded. To achieve taxonomic identification, all sequences (except those recovered in the negative controls) were searched against GenBank’s nucleotide database (*blastn*) using the remote command line interface. For each sequence, the top ten matches were written to a tsv file, but only matches that met the following criteria were included in downstream analyses: 1) a minimum of 90% of the query sequence present in the subject sequence returned from the *blastn* search (*i*.*e*., sequence coverage), 2) at least 95% of overlapping nucleotides being identical (*i*.*e*., sequence identity), 3) an e-value of less than 0.001, and 4) the subject sequence was derived from *ITS2* of a land plant (to rule out inclusion of fungal *ITS2* contaminant sequences). To obtain detailed taxonomic information for each match, the taxid output from *blastn* was used as input for analysis in taxize [22]. For each primer pair, taxonomic abundance charts (TAC) were created in Tableau Desktop v2019.1.0 (Tableau Software Inc., Seattle, WA). To achieve a balance with respect to specificity and informativeness, TAC charts were created at both the order and family level using total read counts and also total sequence counts. Only sequences where 100% congruence amongst the best top matches at either the order or family level were included in any of the TAC. Unique target sequences for ITS2F/ITSp4 and ITSp3/ITSu4 used in TAC generation are available via FigShare (10.6084/m9.figshare.12034848 and 10.6084/m9.figshare.12037155, respectively).

## Results and discussion

### ITS2 primer pair selection and optimization

**Fig 1:**
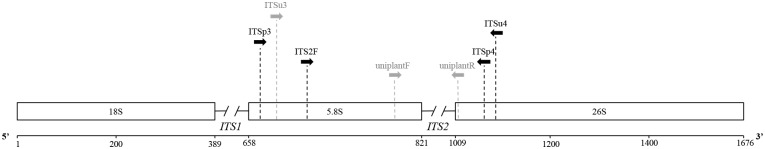
Schematic diagram of binding sites for second internal transcribed spacer (*ITS2*) primers used in this study. Arrow depicts primer direction and flanking regions also shown. Primers shown in light gray indicate those used in screening only; primers shown in black indicate those used in optimization studies.

The *ITS2* primer pairs chosen for testing in this study were as follows: ITS2F/ITSp4 [7,16], ITSp3/ITSu4 [16], uniplantF/uniplantR [17], and ITSu3/ITSu4 [16]. Based on the known primer binding sites (Fig 1), *ITS2* amplicons generated with any of these primer pairs should not exceed 460 bp making them suitable for sequencing on most NGS platforms (S2 Table). A detailed list of the cycling conditions tested in the initial screening of these four primer pairs is given in S2 Table. For ITSu3/ITSu4 and uniplantF/uniplantR, double bands, smearing or complete amplicon drop out was observed using the published cycling conditions and could not be improved with small modifications to the annealing temperature (S2 Table). As *ITS2* can vary in length, typically ~180–390 bp in plants [17], amplicons of differing length were expected between the four plant controls. However strong double bands and smearing was not expected given the controls were derived from single source material, and such results were viewed as indicative of fungal co-amplification. ITS2F/ITSp4 and ITSp3/ITSu4 were selected for further optimization as clean bands of the expected size were mostly generated for each of the four plant control samples. While some smearing in the soil sample was observed using both ITS2F/ITSp4 and ITSp3/ITSu4, this was expected given DNA from multiple plant species was likely isolated.

A shorter extension (20 seconds *vs*. 1 min) eliminated the amplification of multiple products (*i*.*e*., double bands) observed in some samples using ITSp3/ITSu4. A 4% final concentration of DMSO was successful in removing non-specific amplicons for ITS2F/ITSp4, but the addition of DMSO caused PCR failure for some samples using ITSp3/ITSu4 (S3 Table). Thus, the final optimized reaction mix included DMSO at a final concentration of 4% only for ITS2F/ITSp4 and cycling for both primer pairs was as follows: 94°C for 4 min; 40 cycles of 94°C for 30 sec, 55°C for 40 sec and 72°C for 20 sec; and 72°C for 10 min. Clean bands of the expected size were generated when these optimized conditions were used to individually amplify the additional seven single source plant samples.

### Barcode sequence and taxonomic group recovery

Amplification utilizing the optimized conditions for ITS2F/ITSp4 and ITSp3/ITSu4 was successful for the 17 soil samples, as amplicons in the expected size range were observed via gel electrophoresis. Across 38 samples (including positive and negative controls), a total of 13,777,243 raw indexed reads passed filtering from the single MiniSeq sequencing run. After processing through DADA2, the number of reads retained was comparable between primer pairs, however the number of unique sequences recovered was more than double for ITSp3/ITSu4 (Table 1). Only 7.5% of ITSp3/ITSu4 and 43.6% of ITS2F/ITSp4 unique sequences remained for taxonomic comparisons after applying the inclusion criteria for identifying only high-quality plant *ITS2* sequences (see Materials and methods for details) (Table 1). This result indicates that the number of unique sequences output from DADA2 cannot be used to infer experimental success, with respect to sequence quality or target specificity. In the limited previous DNA metabarcoding studies that targeted *ITS2* for plant characterization from soil, larger numbers of target sequences per sample were recovered (*e*.*g*., ~10,000 sequences/sample) [4,23]. Two main differences in methodology can explain the increased recovery in those studies: 1) soil was stored at -80°C prior to subsampling to preserve the biological material [4], and 2) either multiple DNA extractions were completed per sample (with the resulting DNA pooled to increase input for PCR; [4]), or a single extraction was completed using a large quantity of soil as input (4 gm; [23]). Within a primer pair, a similar number of unique plant *ITS2* sequences were available for TAC generation at both the order and family level (Table 1). The number of orders and families recovered per primer pair did differ; ITS2F/ITSp4 consistently outperformed ITSp3/ITSu4 by at least 15% (Table 1). It should be noted that for three samples (ITS2F/ITSp4 = n, 1; ITSp3/ITSu4 = n, 2), no unique plant *ITS2* sequences were retained after applying the inclusion criteria for downstream analyses.

**Table 1:** Comparison of the number of *ITS2* barcode sequences remaining at different stages of data processing and analysis from the 17 soil samples.

		ITS2F/ITSp4	ITSp3/ITSu4
Data processing and filtering*	1) Total unique sequences (reads) from DADA2	2,551 (1,278,943)	5,268 (1,430,676)
	2) Total unique sequences after^#^		
	a) exclusion of sequences not containing the sequence of an *ITS2* primer	1,623 (63.6%)	4,782 (90.7%)
	b) exclusion of duplicates	1,486 (58.3%)	3,688 (70.0%)
	3) Total unique sequences that met *blastn* inclusion criteria^+^^‡^ and		
	a) matched to fungus	101 (6.8%)	404 (11.0%)
	b) matched to plant	648 (43.6%)	277 (7.5%)
*Order level taxon abundance chart*	Total unique sequences used^^^	556	235
	Total number of orders recovered	29	25
	Average (± SD) unique sequences/sample	17 ± 24	15 ± 18
	Average (± SD) reads/sample	8326 ± 9653	5867 ± 6417
*Family level taxon abundance chart*	Total unique sequences used^^^	549	227
	Total families recovered	40	29
	Average (± SD) unique sequences/sample	17 ± 24	9 ± 12
	Average (± SD) reads/sample	8209 ± 9642	5499 ± 5963

*sequences and reads present in the negative controls were excluded;

^#^ percentage calculated based on values shown in 1;

^+^ sequences with >90% sequence coverage, >95% sequence identity, e-value <0.001 and matched to *ITS2*;

^‡^ percentage calculated based on values shown in 2b;

^^^ only sequences in which 100% congruence amongst the best top matches at either the order or family level were included.

To assess the specificity of the optimized conditions, we examined the proportion of high-quality sequences (*i*.*e*., those meeting the *blastn* matching criteria, outlined in the Materials and methods) that were indicative of fungal co-amplification. With either primer pair, the level of fungal co-amplification was <11% (Table 1). One of the well reported disadvantages of using *ITS2* for plant DNA metabarcoding is the high likelihood that fungal DNA will be co-amplified with plant DNA (*e*.*g*., [8,14,16]). This issue is exacerbated in environmental samples such as soil, where fungi are abundant and play a pivot role in decomposition. Further, the abundance of fungi in soil is highly influenced by soil pH; up to a 30-fold increase in fungal growth has been reported in acidic soils (pH ~4.5) [24]. In this study, we did not observe a correlation with the number of fungal *ITS2* sequences recovered and soil pH (r = 0.124 for ITS2/ITSp4 and r = 0.184 for ITSp3/ITSu4).

## Differing approaches to taxonomic abundance chart generation

Considering some previous studies have suggested that read abundance cannot reliably be used as a proxy for the relative natural abundance of plants in a sample (*e*.*g*., [25–30]), we compared the taxonomic composition observed when total read counts and total sequence counts were used to generate TAC. In this study, the most appropriate sample for this comparison would be the pooled positive control, given angiosperm, fern, gymnosperm and moss DNA were pooled for sequencing in equal amounts. For the pooled positive control, total read count and total sequence count TACs were mostly consistent for both primer pairs (S1–S4 Figs). When comparing these two TACs for the soil samples, irrespective of the primer pair or taxonomic level examined, we observed some samples in which the two TACs were fairly consistent (*e*.*g*., ITS2F/ITSp4 sample 16 and 17 at the order level; S1 Fig) and others where the TACs appeared completely different (*e*.*g*., ITSp3/ITSu4 sample 4 at the order level; S2 Fig). We did not note a trend with respect to the number of reads or sequences and TAC similarity (*i*.*e*., samples with higher read/sequence counts did not always have consistent TACs and vice versa; see S4 and S5 Tables for the number of reads and sequences per primer pair and sample). With the small sample size in this study, but also given we do not know the truth plant composition of the surface soils at the time of collection, it is difficult to conclude that one approach for TAC generation outperforms another. To standardize comparisons in this study, data analyses reported subsequently herein are based on the total read count considering more data is available for comparisons (>5500 reads/sample vs >9 sequences/sample).

### Amplification bias using ITS2 primer pairs

An ongoing challenge with using a single primer pair to characterize plant taxa in a bulk sample types is bias in the amplification of certain taxonomic groups over others. While amplification of *ITS2* has been evaluated *in situ* and/or *in vivo* for a wide range of single source land plants (*e*.*g*., [7–9,16,17,31–33]), published evaluations using bulk samples are limited [4,17]. Given *ITS2* was amplified from the same samples using the optimized conditions for ITS2F/ITSp4 and ITSp3/ITSu4, this study represents a unique opportunity to assess whether the primer pair used biases the recovery of certain taxonomic groups. To complete this assessment, first and foremost examination of the pooled positive control is warranted. Notably, no sequences derived from fern DNA (Polypoidales—order, Nephrolepidaceae—family) were recovered using either primer pair (S1–S4 Figs). Whilst Chen and colleagues [7] reported low *ITS2* amplification success for ferns, we anticipated recovery of some fern *ITS2* sequences given amplification with both primer pairs was successful using single source fern DNA. A strong bias for the *Gerbera* (angiosperm DNA) was apparent with both primer pairs; >90% of reads were correctly recovered from Asterales (order) or Asteraceae (family) (S1–S4 Figs).

**Fig 2:**
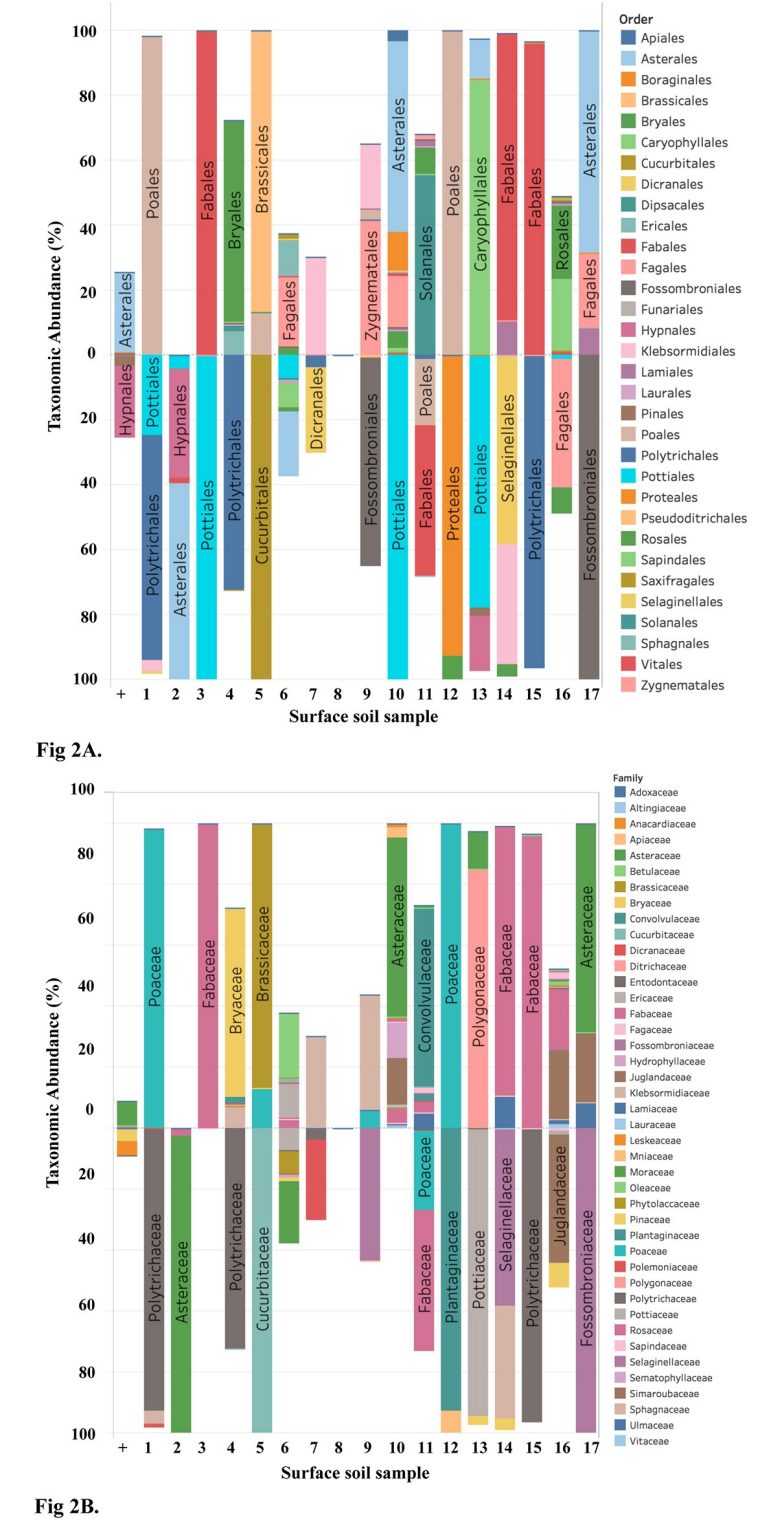
Difference in the taxonomic abundance recovered using ITS2F/ITSp4 (top panes) and ITSp3/ITSu4 (bottom panes) primer pairs. (A) orders. (B) families. Plots generated using total number of recovered reads per surface soil sample.

To assess primer pair amplification bias with bulk environmental samples, previous studies have compared the taxonomic composition recovered using DNA metabarcoding to a plant community survey from the study site (*e*.*g*., [34]). This approach was not possible in this study, given the plant community was not surveyed by the USGS when the soil samples were collected. Whilst county level plant occurrence information is available from the U.S Department of Agriculture via their PLANTS database [35], using PLANTS to backwardly infer plant communities for study sites could be problematic as it a) does not have distribution information for all counties, and b) only contains information about vascular plants, lichens, mosses, liverworts and hornworts that occur naturally, such that introduced species would not be captured [35]. Considering this, in this study primer pair bias was assessed by comparing the taxonomic composition recovered between the two *ITS2* primer pairs. As the same template DNA was used in PCR for both primer pairs, any observed variation would be indicative of primer pair bias. For the majority of the 17 surface soil samples, the taxonomic composition varied quite substantially between primer pairs (Fig 2A and 2B). A bias in the recovery of certain taxonomic groups was observed; ITS2F/ITSp4 preferentially recovered flowering plants (Fabales—order, Fabaceae—family) and grass (Poales—order, Poaceae—family), whereas ITSp3/ITSu4 recovered more moss (Pottiales, Polytrichales—order, Pottiaceae, Polytrichaceae—family) (Fig 2A and 2B). Differing taxonomic recovery could not be linked to sample attributes, for example, higher discordance was not observed exclusive for more acidic soils, or for samples from locations with higher annual rainfall or temperature (S1 Table). Further, unlike in previous studies, we did not observe a bias with the recovery of shorter *ITS2* sequences [17]; over 90% of sequences for both primer pairs were >280 bp (maximum sequence length possible with MiniSeq is 300 bp).

## Conclusions

This study was focused on identifying an appropriate *ITS2* primer pair for broadly characterizing land plants present in soil. Using the cycling conditions and PCR constituents for ITS2F/ITSp4 and ITSp3/ITSu4 optimized in this study, land plants present in 17 soils collected from across the U.S. were characterized. Despite ITS2F/ITSp4 capturing more diversity at both the order and family level, the taxonomic groups recovered from the same sample differed depending on the primer pair used. To address this amplification bias, we advocate that studies using *ITS2* to characterize land plants from bulk material do so using both ITS2F/ITSp4 and ITSp3/ITSu4. Combining the resulting data for a given sample would provide the most accurate representation of the land plant community. Future studies should assess whether the optimized conditions reported in this study are appropriate for characterizing land plants from other bulk environmental sample types.

## Supporting information

S1 Fig Order taxonomic abundance chart for 17 U.S. surface soil samples generated from the total *ITS2* reads (top) and total *ITS2* sequences (bottom) generated using ITS2F/ITSp4. (PDF)

S2 Fig Order taxonomic abundance chart for 17 U.S. surface soil samples generated from the total *ITS2* reads (top) and total *ITS2* sequences (bottom) generated using ITSp3/ITSu4. (PDF)

S3 Fig Family taxonomic abundance chart for 17 U.S. surface soil samples generated from the total *ITS2* reads (top) and total *ITS2* sequences (bottom) generated using ITS2F/ITSp4. (PDF)

S4 Fig Family taxonomic abundance chart for 17 U.S. surface soil samples generated from the total *ITS2* reads (top) and total *ITS2* sequences (bottom) generated using ITSp3/ITSu4. (PDF)

S1 Table Collection and attribute information for the 17 surface soil samples acquired from the USGS [18] collection for use in this study. ^ denotes the sample number assigned for use in figures.
(PDF)

S2 Table Details and subsequent outcome for optimization of cycling conditions for the amplification of *ITS2* using various primer pairs. Bold face denotes conditions selected.
(PDF)

S3 Table Details and subsequent outcome for optimization of PCR constituents for the amplification of *ITS2* using ITS2F/ITSp4 and ITSp3/ITSu4. (PDF)

S4 Table Total number of *ITS2* reads recovered for each sample and used in downstream statistical analyses. (PDF)

S5 Table Total number of *ITS2* sequences recovered for each sample and used in downstream statistical analyses. (PDF)
