# Supplementary figures and images for: Optimization of the second internal transcribed spacer (ITS2) for characterizing land plants from soil
Source: PLoS One. 2020 Apr 16;15(4):e0231436. doi: 10.1371/journal.pone.0231436 (PMC7162488; doi:10.1371/journal.pone.0231436)

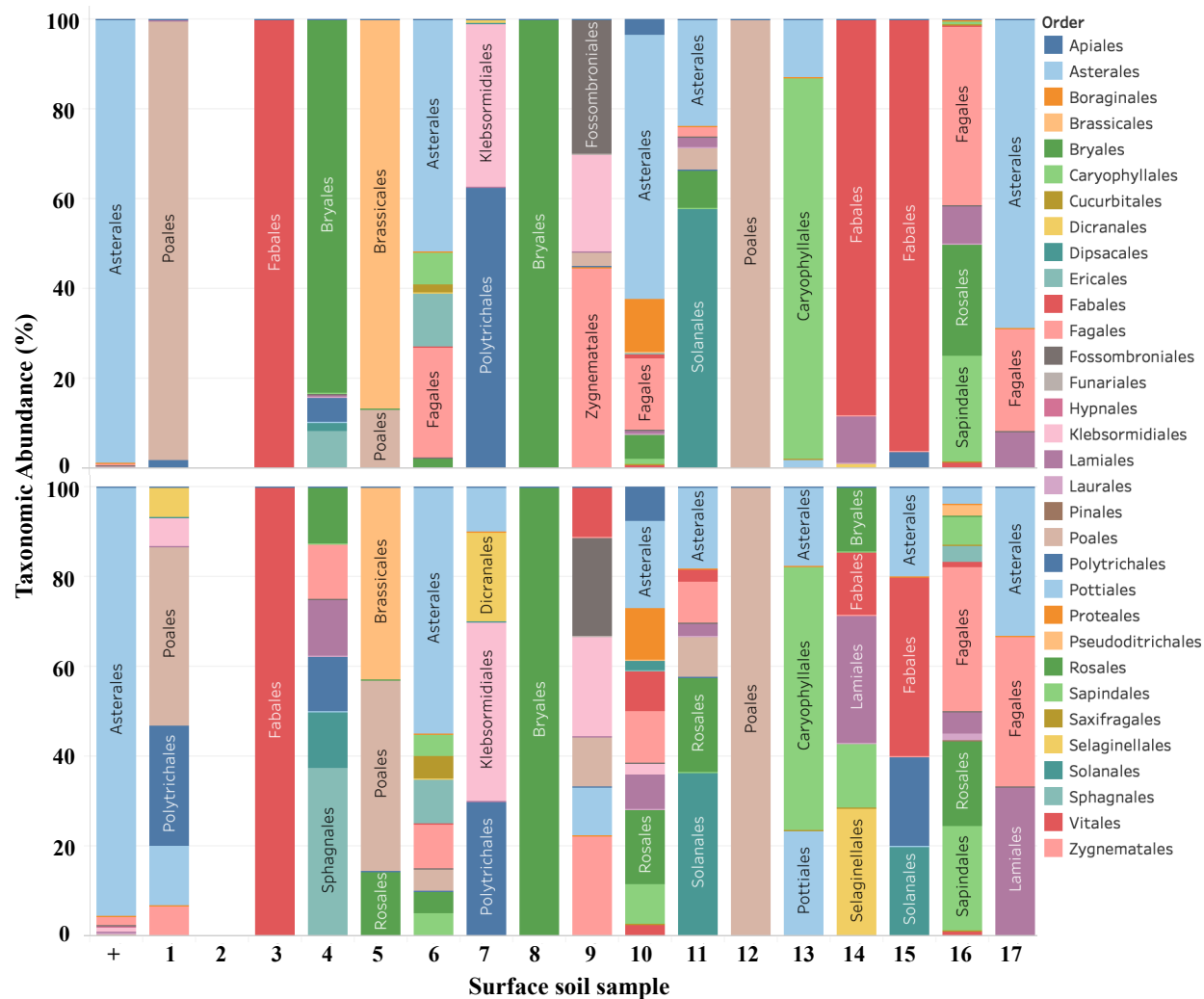

S1 Fig.

Supplement: S1 Fig — (PDF) [file pone.0231436.s001.pdf]

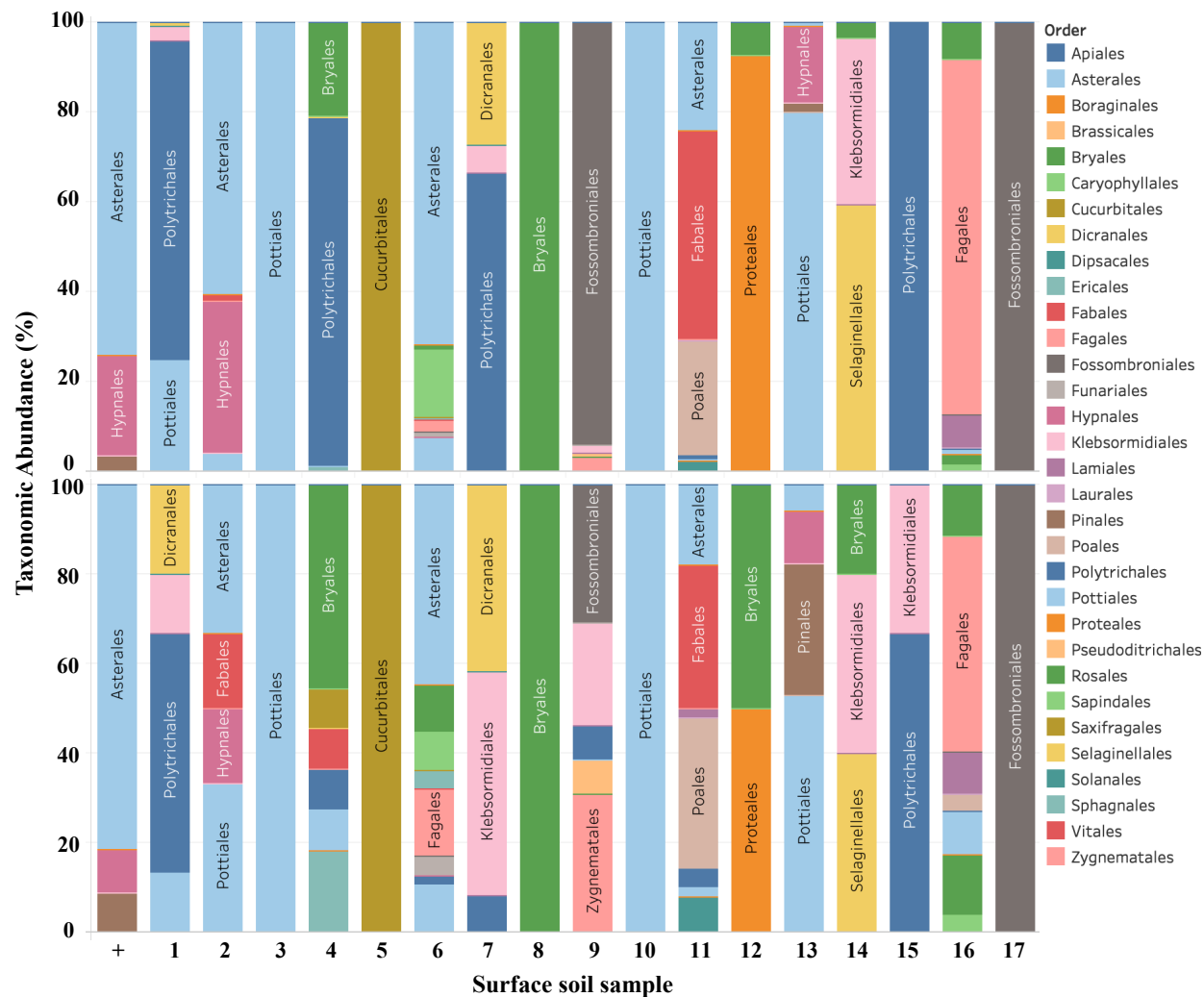

S2 Fig.

Supplement: S2 Fig — (PDF) [file pone.0231436.s002.pdf]

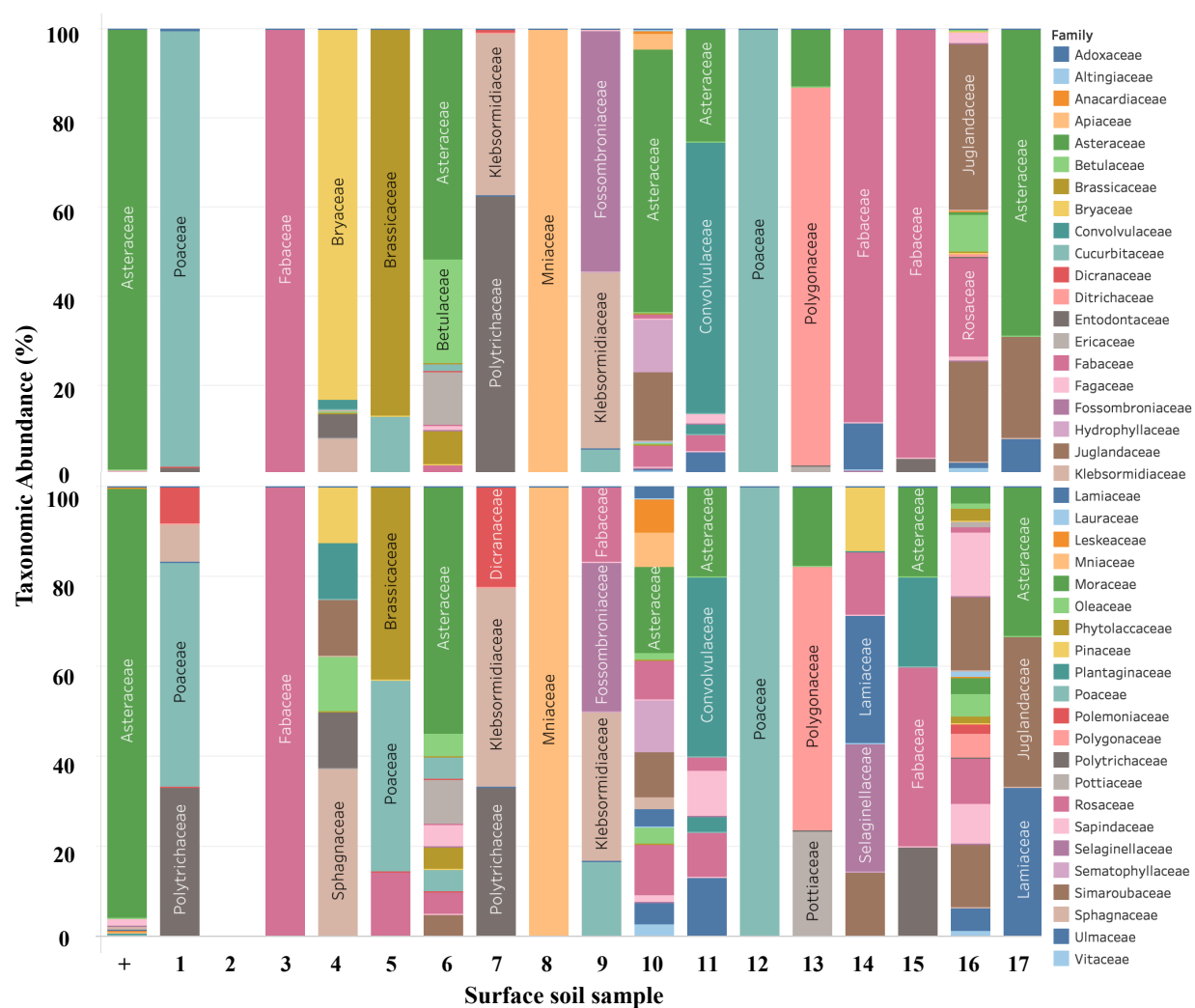

S3 Fig.

Supplement: S3 Fig — (PDF) [file pone.0231436.s003.pdf]

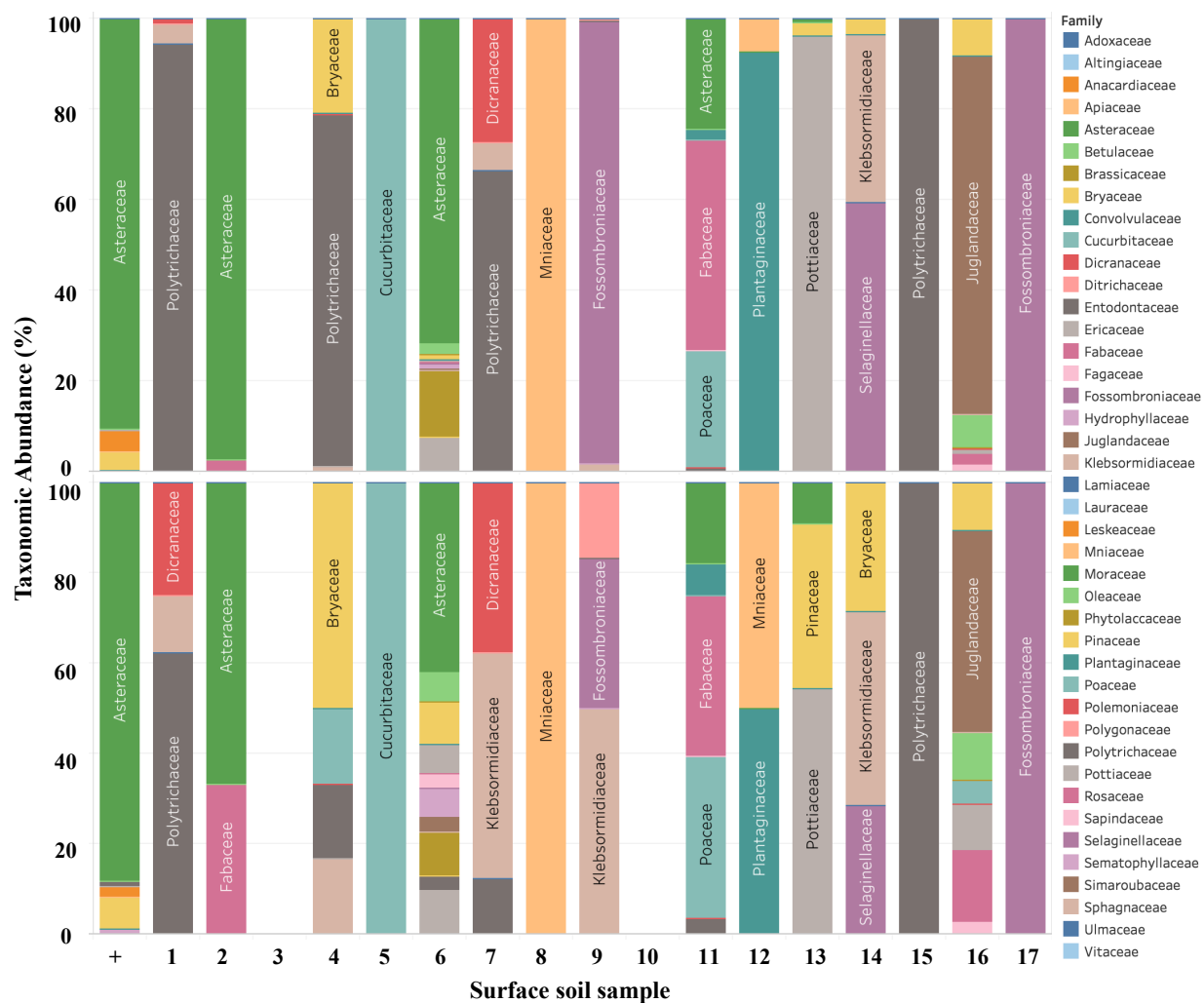

S4 Fig.

Supplement: S4 Fig — (PDF) [file pone.0231436.s004.pdf]
