# Supplementary material for: Optimization of the second internal transcribed spacer (ITS2) for characterizing land plants from soil
Source: PLoS One. 2020 Apr 16;15(4):e0231436. doi: 10.1371/journal.pone.0231436 (PMC7162488; doi:10.1371/journal.pone.0231436)
Supplement: S1 Table — ^ denotes the sample number assigned for use in figures. (PDF) [file pone.0231436.s005.pdf]

S1 Table.

Sample code^	USGS ID Code	Lat	Long	Collection location	pH	Average temperature (^oC)	Average rainfall (mm)	Habitat	Vegetation
1	C-341241	40.7378	-78.415	Allemans, PA	4.5	49	42.44	Forested Upland	Deciduous Forest
2	C-320949	39.8906	-74.3766	Lacey Township, NJ	4.7	53.05	48.78	Forested Upland	Evergreen Forest
3	C-355100	46.0836	-89.9895	Powell, WI	4.8	38.9	32.06	Forested Upland	Mixed Forest
4	C-299988	41.4058	-72.1797	Waterford, CT	4.9	50.55	54.75	Forested Upland	Deciduous Forest
5	C-354779	46.2294	-88.9292	Elmwood, MI	5	45.35	33.12	Forested Upland	Mixed Forest
6	C-350927	35.9649	-81.5831	Warrior, NC	5.3	57.7	47.68	Forested Upland	Deciduous Forest
7	C-299982	42.2245	-72.236	Warren, MA	5.6	47.8	48.02	Forested Upland	Mixed Forest
8	C-320767	38.729	-81.5992	Statts Mills, WV	5.6	54.8	44.02	Forested Upland	Deciduous Forest
9	C-300026	45.1445	-68.8732	Bradford, ME	5.9	44.15	41.91	Forested Upland	Mixed Forest
10	C-349936	39.6019	-81.795	McConnelsville, OH	6.1	51.6	38.28	Forested Upland	Deciduous Forest
11	C-319677	40.1475	-105.932	Granby, CO	6.2	37.3	19.8	Shrubland	Shrubland
12	C-300438	41.1616	-73.8869	Ossining, NY	6.2	50.05	42.01	Forested Upland	Evergreen Forest
13	C-326417	35.091	-97.012	Tribbey, OK	6.2	61.5	36.46	Planted/Cultivated	Pasture/Hay
14	C-311293	39.7916	-94.3832	Osborn, MO	6.3	56.7	39.09	Planted/Cultivated	Row Crops
15	C-364321	37.4717	-79.3258	Lynchburg, VA	6.5	55.5	41.62	Forested Upland	Deciduous Forest
16	C-351841	31.7555	-109.7459	Elfrida, AZ	7.8	70.9	11.92	Planted/Cultivated	Fallow
17	C-313079	37.2557	-110.9821	Lake Powell, UT	7.9	50.6	12.87	Shrubland	Shrubland
