# Supplementary material for: Optimization of the second internal transcribed spacer (ITS2) for characterizing land plants from soil
Source: PLoS One. 2020 Apr 16;15(4):e0231436. doi: 10.1371/journal.pone.0231436 (PMC7162488; doi:10.1371/journal.pone.0231436)
Supplement: S2 Table — Bold face denotes conditions selected. (PDF) [file pone.0231436.s006.pdf]

S2 Table.

Primer pair	Maximum amplicon size	Attempt 1	Outcome	Attempt 2	Outcome	Attempt 3	Outcome
**ITS2F/ITSp4**	400 bp	1X 94°C for 5 min<br>34X 94°C for 30 sec, 55°C for 40 sec, 72°C for 1 min<br>1X 72°C for 10 min	Clean bands (~400-500 bp) for angiosperm, gymnosperm, moss and fern; soil control smeared.	1X 94°C for 4 min<br>5X 94°C for 30 sec, 48°C for 40 sec, 72°C for 1 min<br>35X 94°C for 30 sec, 55°C for 40 sec, 72°C for 1 min<br>1X 72°C for 10 min	Clean faint bands (~400-500 bp) for angiosperm, gymnosperm, moss; fern and soil controls smeared.	**1X 94°C for 4 min**<br>**40X 94°C for 30 sec, 55°C for 40 sec, 72°C for 20 sec**<br>**1X 72°C for 10 min**	Clean strong bands (~400-500 bp) for angiosperm, gymnosperm, moss and fern; soil control smeared.
**ITSp3/ITSu4**	450 bp	1X 94°C for 4 min<br>34X 94°C for 30 sec, 55°C/58°C/61°C for 40 sec, 72°C for 1 min<br>1X 72°C for 10 min	55°C/58°C/61°C - clean strong bands (~400 bp) for angiosperm, gymnosperm, moss and soil; fern no band.	1X 94°C for 4 min<br>40X 94°C for 30 sec, 55°C for 40 sec, 72°C for 1 min<br>1X 72°C for 10 min	Clean bands (~400 bp) for angiosperm, gymnosperm, moss and soil; fern with double bands (~400-500 bp).	**1X 94°C for 4 min**<br>**40X 94°C for 30 sec, 55°C for 40 sec, 72°C for** 5/10/**20**/30 **sec**<br>**1X 72°C for 10 min**	5/10/20/30 sec - Clean bands (~400 bp) for all 5 controls.
**uniplantF/uniplantR**	460 bp	1X 95°C for 10 min<br>40X 95°C for 30 sec, 56°C for 30 sec, 72°C for 1 min<br>1X 72°C for 10 min	Clean bands (~400 bp) for angiosperm and pine; moss, fern no band; soil control smeared.	1X 95°C for 10 min<br>5X 95°C for 30 sec, 48°C for 30 sec, 72°C for 1 min<br>35X 95°C for 30 sec, 56°C for 40 sec, 72°C for 1 min<br>1X 72°C for 10 min	Clean bands (~400 bp) for angiosperm and pine; moss, fern and soil control no band.		
**ITSu3/ITSu4**	410 bp	1X 94°C for 4 min<br>34X 94°C for 30 sec, 55°C/58°C/61°C for 40 sec, 72°C for 1 min<br>1X 72°C for 10 min	55°C - relatively clean bands (~500 bp) all controls. 58°C - clean band (~400 bp) for angiosperm, gymnosperm, moss; fern and soil control weak and smeared. 61°C - smearing in all controls.	1X 94°C for 4 min<br>40X 94°C for 30 sec, 55°C for 40 sec, 72°C for 1 min<br>1X 72°C for 10 min	Double bands (~400-500 bp) in gymnosperm, moss, fern and soil controls; angiosperm clean band (~400 bp).	1X 94°C for 4 min<br>40X 94°C for 30 sec, 55°C for 40 sec, 72°C for 5/10/20/30 sec<br>1X 72°C for 10 min	5 sec - clean single band (~400 bp) for all 5 controls (fern weak). 10, 20 & 30 sec - clean single band (~400 bp) for angiosperm, gymnosperm, moss and soil; fern with double bands (~400-500 bp).
