# Supplementary material for: Optimization of the second internal transcribed spacer (ITS2) for characterizing land plants from soil
Source: PLoS One. 2020 Apr 16;15(4):e0231436. doi: 10.1371/journal.pone.0231436 (PMC7162488; doi:10.1371/journal.pone.0231436)
Supplement: S3 Table — (PDF) [file pone.0231436.s007.pdf]

S3 Table.

Primer pair	Cycling conditions	Final Concentration DMSO	Outcome
**ITS2F/ITSp4**	1X 94°C for 4 min<br>40X 94°C for 30 sec, 55°C for 40 sec, 72°C for 20 sec<br>1X 72°C for 10 min	1.0%	Clean bands (~400 bp) for all 5 controls.
		3.0%	Clean bands (~400 bp) for all 5 controls (fern weak).
		3.5%	Clean bands (~400 bp) for all 5 controls (fern weak).
		4.0%	Clean strong bands (~400 bp) for all 5 controls.
		5.0%	Clean bands (~400 bp) for angiosperm, gymnosperm, fern and soil; moss dropped out.
**ITSp3/ITSu4**	1X 94°C for 4 min<br>40X 94°C for 30 sec, 55°C for 40 sec, 72°C for 20 sec<br>1X 72°C for 10 min	1.0%	Clean bands (~400 bp) for angiosperm and soil; gymnosperm, moss and fern dropped out.
		3.0%	All samples dropped out.
		5.0%	Clean weak bands (~400 bp) for angiosperm and moss; gymnosperm, fern and soil dropped out.
