# Supplementary material for: Optimization of the second internal transcribed spacer (ITS2) for characterizing land plants from soil
Source: PLoS One. 2020 Apr 16;15(4):e0231436. doi: 10.1371/journal.pone.0231436 (PMC7162488; doi:10.1371/journal.pone.0231436)
Supplement: S4 Table — (PDF) [file pone.0231436.s008.pdf]

S4 Table.

Sample number	*Order*		*Family*	
	**ITS2F/ITSp4**	**ITSp3/ITSu4**	**ITS2F/ITSp4**	**ITSp3/ITSu4**
**1**	19023	20811	19023	20811
**2**	12630	11247	12630	9327
**3**	1568	6231	1568	6231
**4**	96	3	96	3
**5**	11	3879	11	3879
**6**	2411	4465	1328	4315
**7**	9763	9227	9763	9227
**8**	3131	354	3131	354
**9**	14202	13728	14202	13728
**10**	0	119	0	74
**11**	18112	5608	17222	5577
**12**	32766	6	32766	0
**13**	5	67	5	67
**14**	19975	16641	19951	12533
**15**	4816	2820	4816	2820
**16**	8	5	8	0
**17**	3037	4533	3037	4533
