# Supplementary material for: Optimization of the second internal transcribed spacer (ITS2) for characterizing land plants from soil
Source: PLoS One. 2020 Apr 16;15(4):e0231436. doi: 10.1371/journal.pone.0231436 (PMC7162488; doi:10.1371/journal.pone.0231436)
Supplement: S5 Table — (PDF) [file pone.0231436.s009.pdf]

S5 Table.

Sample number	*Order*		*Family*	
	**ITS2F/ITSp4**	**ITSp3/ITSu4**	**ITS2F/ITSp4**	**ITSp3/ITSu4**
**1**	78	52	78	38
**2**	17	17	0	11
**3**	8	11	30	6
**4**	3	1	78	1
**5**	1	2	1	1
**6**	9	13	12	6
**7**	7	5	5	3
**8**	7	10	1	7
**9**	20	47	9	31
**10**	0	6	17	3
**11**	33	50	8	28
**12**	78	1	3	0
**13**	1	2	1	2
**14**	15	15	6	8
**15**	5	3	7	2
**16**	1	1	7	0
**17**	10	12	20	8
